# Feasibility and Effectiveness of 3D Coil Framing in the Embolization of Pulmonary Arteriovenous Malformations and Visceral Artery Aneurysms and Pseudoaneurysms

**DOI:** 10.3390/medicina62071298

**Published:** 2026-07-05

**Authors:** Jaeyoon Kim, Yoojin Nam, Pa Hong, Hyeree Cho, Yangwon Kim

**Affiliations:** Department of Radiology, Samsung Changwon Hospital, Sungkyunkwan University School of Medicine, Changwon 51353, Republic of Korea

**Keywords:** three-dimensional coil, coil embolization, pulmonary arteriovenous malformation, splenic artery aneurysm, hepatic artery pseudoaneurysm, visceral aneurysm, endovascular treatment, interventional radiology

## Abstract

*Background and Objectives*: Three-dimensional (3D) coils offer stable initial framing in intracranial aneurysm embolization, but their utility in pulmonary and visceral vascular lesions has not been well established. The objective of this study was to assess the feasibility, safety, and effectiveness of 3D coils used as the primary framing device in pulmonary and visceral embolization. *Materials and Methods*: This retrospective study included 13 patients with 14 lesions, comprising seven pulmonary arteriovenous malformations (PAVMs), six splenic artery aneurysms (SAAs), and one hepatic artery pseudoaneurysm (HAP), treated between 2024 and 2025. In all lesions, a 0.018-inch 3D coil was deployed as the initial framing coil, followed by further packing with additional 3D or non-3D coils. Technical success, angiographic success, and clinical outcomes were evaluated. *Results*: Technical success was achieved in all lesions (14/14, 100%; 95% CI, 78.5–100). Angiographic success was achieved in 13 of 14 lesions (92.9%; 95% CI, 68.5–98.7), and clinical success was achieved in 12 of 13 lesions (92.3%; 95% CI, 66.7–98.6). Post-embolization syndrome (PES) developed in five patients with SAAs and in one patient with HAP; all complications were minor and resolved with conservative management. No coil migration or major complications occurred. *Conclusions*: When used as the initial framing device, 3D coils were technically feasible and achieved acceptable short-term angiographic and clinical outcomes in this small, heterogeneous series; these preliminary findings require confirmation in comparative studies.

## 1. Introduction

Three-dimensional (3D) coils were developed to enhance initial framing stability, conformability to complex lesion geometry, and packing efficiency because of their pre-shaped configuration, which facilitates stable and controlled coil deployment [1,2,3,4]. Although these advantages have been well demonstrated in the treatment of intracranial aneurysms, their applicability to pulmonary and visceral vascular lesions has not been adequately evaluated.

**Figure 1:**
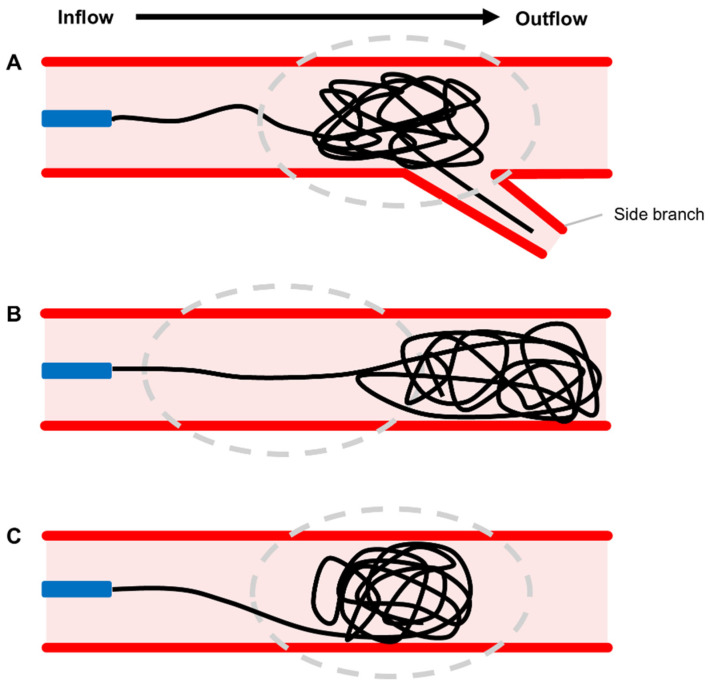
Schematic illustration comparing conventional coil framing with 3D coil framing. The gray dashed outline indicates the intended initial framing target. (**A**) Conventional anchoring technique, in which the coil is stabilized at the target using a side branch. (**B**) When a suitable side branch is unavailable or anchoring is inadequate, the coil is displaced distal to the intended framing target. (**C**) A 3D coil conforms to the target and forms a stable spherical configuration at the intended site without the need for adjunctive anchoring.

Endovascular coil embolization is a well-established treatment for pulmonary arteriovenous malformations (PAVMs), visceral artery aneurysms, and pseudoaneurysms. For PAVMs, transcatheter embolization is the established treatment, with occlusion typically directed at the feeding artery close to the venous sac and/or the sac itself to prevent persistent shunting or reperfusion. In visceral artery aneurysms and pseudoaneurysms, coil embolization is commonly performed using sac packing, inflow–outflow isolation, or a combination of these techniques, depending on lesion morphology, branch anatomy, and the need to preserve distal perfusion. Despite differences in disease entity and embolization strategy, these procedures share a common technical challenge: achieving stable initial coil positioning in high-flow or anatomically complex vascular structures. Failure to obtain stable initial framing or anchoring may result in coil migration and non-target embolization. To enhance initial coil stability, anchoring or scaffolding techniques are commonly used during framing [5,6] (Figure 1). Nevertheless, the technical feasibility of these techniques may differ according to the vascular anatomy.

In this context, the inherently stable 3D configuration of these coils may offer a framing strategy that is less dependent on anatomy, thereby allowing secure and controlled initial coil deployment in challenging lesions. Accordingly, this study aimed to evaluate the technical feasibility, safety, and clinical effectiveness of 3D coils as a primary framing device in pulmonary and visceral vascular lesions, including PAVMs, visceral artery aneurysms, and a pseudoaneurysm.

## 2. Materials and Methods

### 2.1. Study Design and Patient Selection

This retrospective study was approved by the institutional review boards, and the requirement for informed consent was waived. Embolization cases in the interventional suites during a 2-year period (2024–2025) were reviewed. Cases were included if a 3D coil was used as the first deployed coil for framing, with or without subsequent packing, during endovascular embolization. Cases were excluded if a 3D coil was used only as an adjunct during the packing phase or if the target framing segment exceeded the maximum available diameter of the 3D coil system used (Concerto, Medtronic; available in Korea in diameters of 5–10 mm), requiring embolization with larger non-3D coils or other alternative strategies. During this period, 135 transcatheter embolization procedures were performed for various indications, of which 26 targeted a discrete saccular lesion amenable to initial coil framing (an arteriovenous malformation, true aneurysm, or pseudoaneurysm). A 3D coil (Concerto) was selected as the initial framing device, at the operator’s discretion, when the lesion required stable initial framing or precise positioning of the coil at the target because of high flow or unfavorable anatomy or morphology, within the available 3D-coil diameter range (5–10 mm). A 3D coil was used as the initial framing device in 13 of these 26 procedures, which constituted the study cohort (pulmonary arteriovenous malformations, splenic artery aneurysms, and a hepatic artery pseudoaneurysm). The remaining 13 lesions were treated with conventional coils (*n* = 5), a liquid embolic agent (*n* = 4), or gelatin sponge (*n* = 4), generally because they were less amenable to 3D-coil framing, for example, when the target framing diameter fell outside the available 5–10 mm range or the lesion morphology or patient condition favored another technique. The selection process is summarized in Supplementary Figure S1. A total of 13 patients with 14 vascular lesions were included, comprising 7 PAVMs, 6 splenic artery aneurysms (SAAs), and 1 hepatic artery pseudoaneurysm (HAP).

### 2.2. Patient Data Collection

Clinical, procedural, and imaging data were retrospectively collected from electronic medical records and the imaging database. Collected variables included basic patient demographics, clinical information, lesion type and location, procedural details, and treatment outcomes.

### 2.3. Imaging Analysis and Vessel Measurement

Pre-procedural CT was performed on a 128-slice multidetector CT scanner (SOMATOM Definition AS+, SOMATOM Definition Flash, or SOMATOM Force; Siemens Healthineers, Erlangen, Germany) with images reconstructed at 2 mm slice thickness for chest and 3 mm slice thickness for abdominal imaging, with arterial-phase contrast enhancement. Angiography was performed using a digital subtraction angiography system (Allura Clarity Xper 20/50 or Azurion 7 M20; Philips Healthcare, Best, The Netherlands). Pre-procedural contrast-enhanced CT was reviewed to assess lesion morphology and vascular anatomy. For PAVMs, the maximal diameters of the venous sac, feeding artery, and draining vein were assessed using pre-procedural CT and intra-procedural angiographic images. Post-treatment changes in draining vein diameter were evaluated on follow-up imaging. For visceral arterial aneurysmal lesions, the maximal aneurysm sac diameter and the diameters of inflow and outflow branches were assessed using pre-procedural CT and intra-procedural angiographic images. The maximum diameter of the lesions was measured by two radiologists in consensus.

### 2.4. Procedural Technique

All procedures were performed by three interventional radiologists with 3–16 years of experience using standard endovascular techniques under local anesthesia. Vascular access was obtained via femoral arterial or venous routes, depending on lesion type. Despite the heterogeneous lesion types, all embolization procedures followed a shared technical principle: deployment of a 0.018-inch 3D coil (Concerto, Medtronic, Minneapolis, MN, USA) as the first framing device to establish a stable scaffold, followed by coil embolization adapted to lesion-specific anatomy and hemodynamics. After diagnostic angiography, selective catheterization of the target vessel was achieved using a coaxial microcatheter system (Progreat, Terumo, Tokyo, Japan). A 3D coil with a diameter at least 10% larger than the target lesion was deployed as the initial framing coil, allowing it to conform to the lesion and form a stable scaffold for subsequent packing. Additional 0.018-inch 3D or non-3D coils, including helical coils (Concerto, Medtronic, Minneapolis, MN, USA) or fibered coils (Interlock, Boston Scientific, Marlborough, MA, USA), were deployed for further sac packing or embolization of inflow or outflow branches. Adjunctive embolic materials were selectively used at the operator’s discretion based on lesion size, residual flow, and the perceived need for additional embolization.

### 2.5. Lesion-Specific Techniques

#### 2.5.1. PAVMs

**Figure 2:**
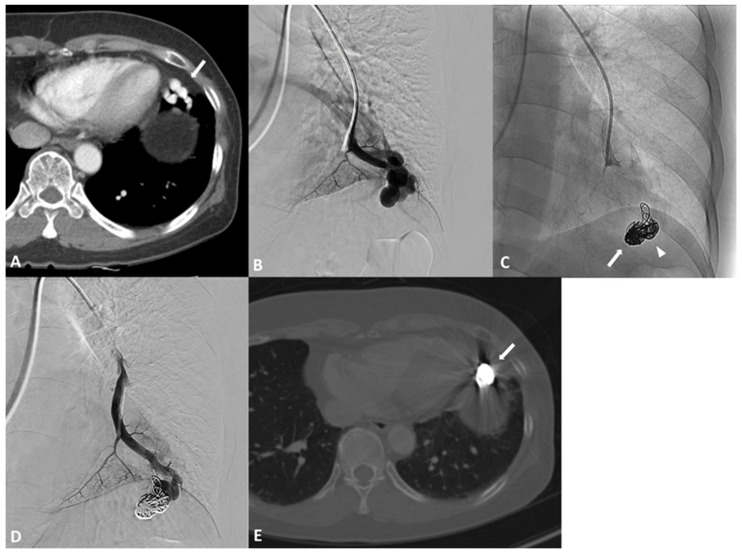
A 62-year-old woman with pulmonary arteriovenous malformation (PAVM). (**A**) Contrast-enhanced chest CT shows a PAVM in the left lower lobe (arrow). (**B**) Selective pulmonary angiography of the feeding artery confirms the lesion. (**C**) A 10 mm 3D coil is placed in the venous sac as the initial framing coil (arrow), followed by additional coils (arrowhead). (**D**) Final angiogram demonstrates complete occlusion of the venous sac, with no visible draining vein. (**E**) Follow-up CT at 3 months shows densely packed coils within the PAVM (arrow) and regression of the draining vein.

Patients received an initial heparin bolus of 2000–4000 units, followed by additional 1000-unit boluses administered hourly. A microcatheter was advanced into the venous sac. A 3D coil was deployed as the initial framing coil, followed by additional 3D or non-3D coils for venous sac packing, with extension into the distal feeding artery to achieve complete occlusion of the sac and feeding artery (Figure 2).

#### 2.5.2. SAAs

**Figure 3:**
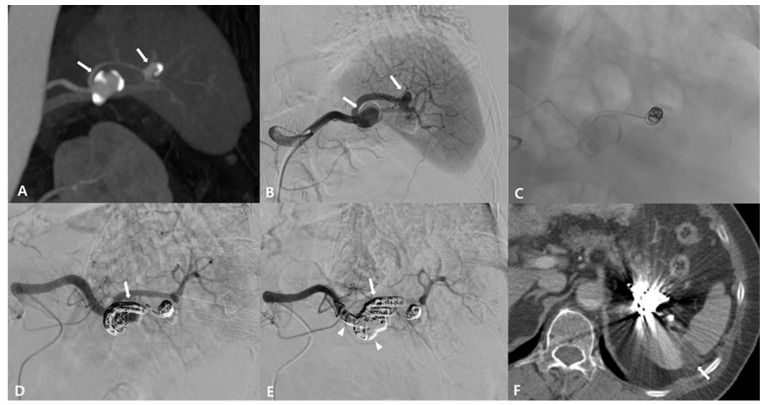
A 75-year-old woman with two splenic artery aneurysms. (**A**,**B**) Maximum-intensity-projection image of contrast-enhanced CT and selective splenic angiography show two SAAs (arrows). (**C**) A 6 mm 3D coil is placed in the smaller aneurysm in the superior polar branch. (**D**) A 5 mm 3D coil is placed in one outflow branch for initial framing (arrow). (**E**) Another 5 mm 3D coil (arrow) is placed in the other outflow branch, followed by additional 3D and non-3D coils placed in the aneurysm sac and inflow branch to achieve complete sac occlusion (arrowhead). (**F**) Follow-up CT at 2 months shows dense coil packing in both SAAs, with partial splenic infarction (arrow).

A microcatheter was advanced into the outflow branch of the splenic artery aneurysm, and a 3D coil was placed in the outflow branch for initial framing. Additional 3D or non-3D coils were then deployed to occlude the outflow branches, aneurysm sac, and inflow branches (Figure 3). In selected cases with large sac volumes, gelatin sponge particles were used as adjunctive embolic material.

#### 2.5.3. HAPs

**Figure 4:**
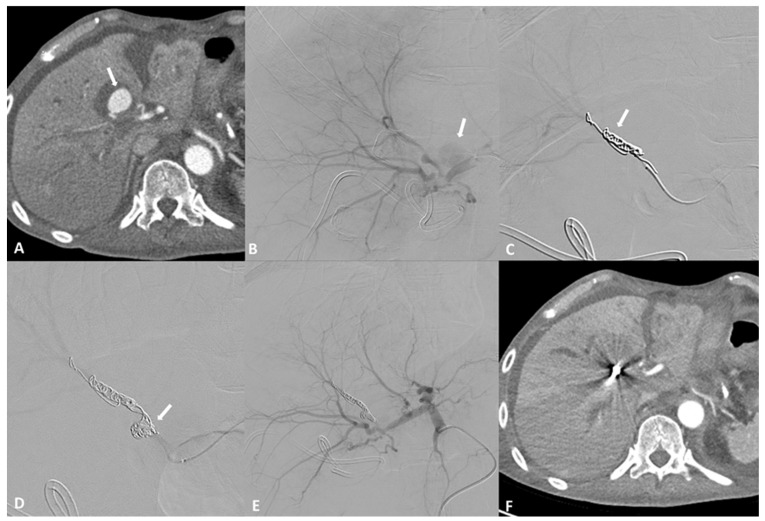
A 50-year-old man with a ruptured hepatic artery pseudoaneurysm (HAP). (**A**) Contrast-enhanced abdominal CT shows a ruptured hepatic artery pseudoaneurysm (arrow). (**B**) Selective hepatic angiography demonstrates a pseudoaneurysm arising from the right anterior hepatic artery (arrow). (**C**) A 5 mm 3D coil is placed in the outflow branch for initial framing (arrow). (**D**) Additional coils are placed in the focal aneurysmal bulge at the pseudoaneurysm origin (arrow) and the inflow branch. (**E**) Final angiography demonstrates complete occlusion of the pseudoaneurysm without further contrast extravasation. (**F**) Follow-up CT at 1 week shows complete embolization of the pseudoaneurysm without evidence of active bleeding.

A microcatheter was advanced distal to the pseudoaneurysm, and a 3D coil was placed in the outflow branch for initial framing, followed by embolization of the focal aneurysmal bulge at the lesion origin and the inflow branch to achieve complete exclusion and hemostasis (Figure 4).

### 2.6. Follow-Up

For PAVMs, follow-up CT was performed 3–6 months after embolization to assess changes in the venous sac and draining vein. For SAAs, follow-up CT was performed at 1–3 months to assess aneurysm sac thrombosis or occlusion and detect recanalization. For the HAP case, follow-up CT was performed at 2 weeks to confirm the complete exclusion of the pseudoaneurysm and the absence of active bleeding. Follow-up imaging was available for 13 of 14 lesions, as one patient was lost to follow-up.

### 2.7. Definitions of Outcomes

Outcomes were assessed on a lesion basis. Technical success was defined as the successful deployment of a 3D coil as the primary framing device without migration or need for repositioning. Angiographic success was defined as complete occlusion of the target lesion with no residual contrast filling on the final angiogram. Partial flow reduction without complete occlusion was classified as angiographic failure. Clinical success was defined as a ≥70% reduction in venous sac and draining vein size on follow-up CT for PAVMs [7,8,9], and as complete sac embolization or absence of rebleeding for visceral artery aneurysms and pseudoaneurysms. Complications were classified as major or minor according to the Society of Interventional Radiology guidelines.

### 2.8. Statistical Analysis

Descriptive statistics were used to summarize lesion characteristics and coil utilization by disease category. Continuous variables, including target lesion size, number of 3D coils, non-3D coils, and total coils, were expressed as mean ± standard deviation with corresponding ranges (minimum–maximum). Outcome analyses were performed on a per-lesion basis, whereas symptom status and post-embolization syndrome (PES) were summarized on a per-patient basis. Binomial success proportions are reported with 95% Wilson score confidence intervals. All statistical analyses were performed using R software (version 4.6.0; R Foundation for Statistical Computing, Vienna, Austria).

## 3. Results

A total of 13 patients (mean age, 60.62 ± 11.22 years; 3 men and 10 women) with 14 lesions were included. The lesions included 7 PAVMs, 6 SAAs (in 5 patients), and 1 HAP (Table 1). Two patients (15.4%) were symptomatic (hemoptysis, *n* = 1; hypotension, *n* = 1), whereas the others were asymptomatic and diagnosed incidentally.

In PAVMs, the mean diameter of the feeding artery was 4.20 ± 1.47 mm (range, 2.8–6.3 mm), the draining vein was 5.30 ± 1.85 mm (range, 3.0–8.3 mm), and the venous sac was 7.49 ± 1.56 mm (range, 5.0–9.1 mm). In SAAs, the mean diameter of the inflow branch was 4.34 ± 1.82 mm (range, 3.1–7.5 mm), the outflow branch was 3.17 ± 0.44 mm (range, 2.6–3.9 mm), and the aneurysm sac was 27.67 ± 22.18 mm (range, 6.0–63.0 mm). In HAP, the inflow branch measured 2.9 mm, the outflow branch 2.8 mm, and the pseudoaneurysm sac 22 mm.

The mean number of 3D coils used per lesion was 3.07 ± 1.27 (range, 1–6). The mean number of non-3D coils (helical and fibered) used per lesion was 4.93 ± 3.83 (range, 1–16), with a mean total of 8.00 ± 3.82 coils per lesion (range, 2–18). The mean total procedure time was 83.2 ± 45.8 min (range, 30–165 min). By lesion type, the mean procedure time was 74.4 ± 58.5 min for PAVMs, 95.6 ± 29.0 min for SAAs, and 83 min for the single HAP (Table 2).

The median imaging follow-up duration among the 13 lesions with available follow-up imaging was 4.5 months (range, 2 weeks to 20.5 months); among the six PAVMs with available follow-up imaging, the median follow-up duration was 8.0 months (range, 3.7–20.5 months). Surveillance imaging was performed with contrast-enhanced CT in all cases.

Technical success was achieved in all 14 lesions (100%; 95% CI, 78.5–100), defined as successful deployment of a 3D coil as the initial framing device without migration or need for repositioning.

Angiographic success was achieved in 13 of 14 lesions (92.9%; 95% CI, 68.5–98.7): 6 of 7 PAVMs (85.7%; 95% CI, 48.7–97.4), 6 of 6 SAAs (100%), and 1 of 1 HAP (100%). Clinical follow-up was available for 13 of 14 lesions, as one patient was lost to follow-up. Clinical success was achieved in 12 of 13 evaluable lesions (92.3%; 95% CI, 66.7–98.6): 5 of 6 PAVMs (83.3%; 95% CI, 43.6–97.0), 6 of 6 SAAs (100%), and 1 of 1 HAP (100%) (Table 3). Because of the small per-lesion denominators, the 95% confidence intervals were wide, particularly in the lesion-specific subgroup estimates.

One PAVM case resulted in angiographic and clinical failure. Severe anatomic difficulty limited guiding catheter advancement, resulting in prolonged procedure time and intentional termination before complete occlusion. The final angiogram showed incomplete flow reduction, and 3-month follow-up CT demonstrated only a 35% decrease in draining vein size.

PES occurred in all patients with SAAs (5/5) and in one patient with HAP (6/13, 46.2%). PES manifested as self-limiting abdominal pain in all affected patients and resolved within 3 days with conservative management; no PES-related event required additional procedures or treatment, and none resulted in prolongation of hospital stay. Splenic infarction was observed on follow-up CT in all 5 patients with SAAs. As no universally accepted imaging criterion for grading splenic infarction exists, the extent was graded by the proportion of splenic parenchyma involved, adapting the approach reported by Yoon et al. [10]: less than one-third in one patient and one-third to two-thirds in four patients, with no infarction involving more than two-thirds of the spleen. The splenic infarctions were accompanied by the self-limiting symptoms of PES described above and were classified as minor complications that resolved without sequelae. No major complications occurred (Table 3).

## 4. Discussion

Prior neurointerventional studies have shown that secure first-coil framing is a key determinant of embolization durability, because inadequate framing may lead to coil migration during subsequent packing or recurrence [2,4]. The mechanical advantages of 3D coils, including framing stability, conformability to complex lesion geometry, and packing efficiency, are highly relevant to pulmonary and visceral vascular lesions, in which high-flow dynamics and irregular anatomy may similarly compromise first-coil stability. To the best of our knowledge, no published studies have specifically examined the use of 3D coils as a primary framing device outside intracranial aneurysm embolization. In the present study, the neurointerventional principle of using a 3D coil as the initial framing device was successfully extended to the embolization of PAVMs, visceral artery aneurysms, and pseudoaneurysms.

Although the inclusion of heterogeneous vascular lesions—PAVMs, SAAs, and HAP—introduces variability in lesion morphology, flow dynamics, and procedural complexity, these lesion types share a common technical challenge: the need for stable initial coil framing in anatomy lacking a well-defined vascular neck or a suitable side-branch anchor. The shared technical principle of deploying a 3D coil as the primary framing scaffold was the rationale for their collective analysis in this preliminary study. Nevertheless, the mechanical advantages of 3D coils may differ according to lesion type. In PAVMs, the spherical 3D configuration may facilitate venous sac filling and reduce the risk of coil prolapse into the feeding artery. In SAAs, the 3D geometry provides outflow-branch stabilization prior to retrograde sac packing. In pseudoaneurysms, it secures the outflow tract before inflow occlusion, mimicking the structural scaffold principle described for other endovascular occlusive devices. Future studies should examine each lesion type separately to clarify lesion-specific technical advantages.

### 4.1. PAVMs

Various embolic materials have been used to treat PAVMs, including coils, vascular plugs, occlusion balloons, and N-butyl cyanoacrylate. Embolization strategies include occlusion of the feeding artery alone, embolization of the venous sac, or combined embolization of the venous sac and feeding artery [7,8,11]. Previous studies have shown that embolization involving the venous sac, with or without extension into the feeding artery, is associated with lower recanalization rates than embolization of the feeding artery alone [7,9,11]. Accordingly, in the present study, coil embolization targeted both the venous sac and the feeding artery.

PAVMs are high-flow lesions in which stable initial framing is critical for subsequent coil embolization, because unstable framing may result in coil migration and non-target embolization. Traditional anchoring and scaffolding techniques have been widely used for coil framing [5,6]. However, advancing the coil tip into a small side branch is not always feasible, and anchoring or scaffolding may at times be achieved at a site more distal or proximal than intended by the operator. In such situations, 3D coils may conform to the lesion geometry and form a spherical configuration that facilitates controlled and stable framing at the intended target location. Beyond preventing migration, precise framing at the intended target may also affect procedural efficiency: when framing is achieved more distally or proximally than intended, unintended segments may be occluded and additional coils may be required to reach the desired endpoint. This is relevant both to high-flow PAVMs and, in visceral artery aneurysms and pseudoaneurysms, to confining occlusion to the intended segment. The self-forming, conformable configuration of 3D coils may help achieve such targeted framing.

Previous studies have generally recommended coil oversizing of at least 20% or 2 mm relative to the venous sac or feeding artery to improve stability in high-flow settings [5,11]. In contrast, Srinivas et al. [8] reported high technical success using coils sized at a 1:1 ratio to the target venous sac and feeding artery, achieving complete occlusion in both short-term (58/58, 100%) and long-term follow-up (17/17, 100%) in patients with simple PAVMs. In the present study, 3D coils were selected with at least 10% oversizing relative to the target lesion, which provided stable framing and an effective scaffold for subsequent coil packing. The angiographic and clinical success in our PAVM subgroup falls within the range reported for conventional coil embolization, in which technical success and durable occlusion generally exceed 85–90% [8,9]; the absence of a comparator precludes any inference of superiority.

Complete angiographic occlusion is the standard procedural endpoint for PAVM embolization. In the single case of angiographic and clinical failure in this study, advancement of the guiding catheter into the feeding artery was technically difficult because of vascular tortuosity. Although a 3D coil was successfully placed as a framing device, the procedure was intentionally stopped before complete occlusion was achieved because of patient intolerance related to the prolonged procedure time. Based on our clinical experience, marked flow reduction in the target lesion may result in delayed complete occlusion on follow-up imaging. However, delayed complete occlusion was not observed in this case, underscoring the importance of achieving complete angiographic occlusion during the procedure.

The most common complication reported after PAVM embolization is transient pleurisy, which occurs in approximately 10% of patients [12]. In this study, no minor or major complications were observed.

### 4.2. Splenic Artery Aneurysms

Endovascular treatment has become the first-line therapeutic option for SAAs, with available strategies including coil embolization with or without inflow or outflow occlusion, stent-assisted coiling, and stent-graft exclusion [13,14,15]. Treatment selection primarily depends on aneurysm size, location, and vascular anatomy, and coil embolization is generally preferred for distal SAAs [13].

When performing coil embolization, careful review of pre-procedural contrast-enhanced CT and intra-procedural angiography is essential because collateral vessels, such as the short gastric and dorsal pancreatic arteries, may contribute to splenic perfusion. In distal SAAs, caution is required to avoid unintended embolization of these collateral branches. In this setting, 3D coils allow precise deployment and stable framing at the intended target site, thereby facilitating controlled embolization and reducing the risk of distal embolization.

All SAAs in our cohort were saccular and located in the distal splenic artery. Coil embolization was therefore selected based on lesion morphology and vascular anatomy. In one patient, a small (6 mm) aneurysm was identified in the superior polar branch, which served as the outflow branch of a larger SAA. Selective embolization was performed despite its small size because of concern about future procedural difficulty if the aneurysm enlarged. In two cases with large aneurysm sacs (47 mm and 63 mm), adjunctive gelatin sponge embolization was used to achieve complete occlusion and reduce recanalization risk.

All patients with SAAs (5/5) developed PES and partial splenic infarction. In a study by Yoon et al., PES and splenic infarction occurred in 55.6% and 84.6% of patients, respectively, after distal splenic artery aneurysm embolization [10]. Furthermore, embolization of the aneurysm with in- and outflow branch occlusion has been reported to have a higher splenic infarction rate than sac embolization [13,16,17]. The predominance of distal embolization and the embolization techniques used in the present study may have contributed to the high incidence of PES and splenic infarction in our patients.

Despite the high incidence of partial splenic infarction, all cases were clinically insignificant and resolved with conservative management, without progression to splenic abscess, rupture, need for splenectomy, or prolonged hospitalization. These outcomes are comparable to the technical success (≈95–100%) and clinical success (~91%) reported for endovascular SAA treatment with conventional techniques [15,16], again without implying superiority in the absence of a comparator.

### 4.3. HAP

HAP is a rare but potentially life-threatening condition. Risk factors for HAP include hepatobiliary and pancreatic procedures, infection, trauma, atherosclerosis, hypertension, liver transplantation, and tumor invasion [18]. In our case, a prior CT scan demonstrated a metastatic lesion involving the porta hepatis and hepatoduodenal ligament, which may have caused hepatic arterial wall injury with subsequent pseudoaneurysm formation.

Endovascular treatment options for HAP include embolization and stent-graft placement. Various embolization techniques have been described, including the sandwich technique, sac packing, proximal occlusion, stent-assisted coiling, and balloon remodeling [19]. In the present case, a stent-graft appropriately sized for the target lesion was not available at the time of the procedure. Therefore, 3D coil embolization was performed with initial framing of the outflow branch, followed by embolization of the focal aneurysmal bulge at the pseudoaneurysm origin and the inflow branch. Framing of the outflow branch provided a stable coil framework, facilitating controlled coil deployment and preventing coil migration during subsequent embolization. Although the sandwich technique alone might have been sufficient, the exact neck origin was not clearly delineated, and a small focal aneurysmal bulge at the pseudoaneurysm origin was considered part of the pseudoaneurysm spectrum. Therefore, embolization was extended to include this segment to ensure complete exclusion of the compromised arterial wall and reduce the risk of recurrence. PES occurred and was managed conservatively without major complications.

The inherent 3D geometry and conformability of 3D coils may enable stable and precise framing at the intended target site in complex vascular lesions. In this series, 3D coils performed reliably as the initial framing device without coil migration or repositioning, and acceptable short-term angiographic and clinical outcomes were observed despite differences in lesion anatomy and flow dynamics. These findings suggest that 3D coils may serve as a technically viable primary framing device in selected embolization procedures. More specifically, this role appears to be technical rather than disease-specific: 3D-coil framing may be particularly relevant when conventional anchoring is difficult, when precise localization of the initial coil is important, or when unintended distal or proximal coil displacement could compromise the intended embolization endpoint. This interpretation should be tested in future lesion-specific comparative studies using objective procedural and durability endpoints.

The concept of 3D coil framing as a structural scaffold that promotes stable thrombosis while preventing migration shares conceptual similarities with other endovascular occlusive devices. Georgiades et al. described successful salvage of an aortic rupture using an Occlutech device that created a stable occlusive scaffold promoting thrombus formation while preserving adjacent vascular structures [20]. This structural framing principle—whereby an initial stable scaffold facilitates controlled thrombosis—may similarly underlie the favorable technical outcomes observed with 3D coils in fistulous and aneurysmal lesions in the present series. From a practical standpoint, in the Korean national health insurance reimbursement system, 3D coils are covered at the same rate as standard helical coils, representing no additional cost burden in this setting. Formal cost-effectiveness evaluation in larger comparative studies will be necessary to determine whether any broader economic advantages exist.

### 4.4. Limitations

This study has several limitations. It was limited by its retrospective single-center design, small sample size (13 patients/14 lesions), and heterogeneous lesion types. Because of the small per-lesion denominators, the success proportions are accompanied by wide confidence intervals and should be regarded as descriptive rather than as precise estimates of effectiveness. The absence of a control group treated with conventional coils precludes direct comparison and limits conclusions regarding relative superiority. Potential selection bias may have influenced lesion selection and device choice, as 3D coils were used at the operator’s discretion. In addition, local device availability restricted 3D coil diameters to 5–10 mm, limiting generalizability to larger lesions. The study primarily focused on technical feasibility, and follow-up imaging was limited to short- to mid-term intervals; therefore, long-term durability could not be assessed. Larger prospective comparative studies are warranted to confirm the clinical advantages of 3D coils over conventional framing techniques. Because surveillance relied on short- to mid-term CT, late recanalization, which may occur months to years after PAVM embolization, could not be excluded; in accordance with the CIRSE Standards of Practice, long-term imaging surveillance beyond the initial 6-month assessment is recommended to detect late reperfusion [21]. The present findings should therefore be interpreted as a short-term feasibility experience rather than as evidence of long-term efficacy. Furthermore, fluoroscopy time and radiation dose were not systematically recorded, and the absence of a comparator group precludes objective assessment of the relative procedural efficiency of 3D-coil framing. Three operators with varying levels of experience (3–16 years) performed all procedures; although a standardized protocol was applied consistently across cases, variability in operator experience may have introduced unmeasured technical bias. Qualitative outcomes, including operator feedback, procedural ease, and device handling characteristics, were not systematically assessed and represent an additional limitation. The most significant limitation is the inclusion of heterogeneous disease entities with substantially different vascular anatomy, hemodynamics, and treatment strategies. PAVMs, SAAs, and HAP represent distinct pathological entities, and pooling them limits the interpretability of the results. This study should therefore be interpreted as a preliminary feasibility experience rather than definitive evidence of efficacy for any individual disease.

## 5. Conclusions

Used as the initial framing device, 3D coils were technically feasible and achieved acceptable short-term angiographic and clinical outcomes in this small, heterogeneous series of pulmonary and visceral vascular lesions. These preliminary, hypothesis-generating findings require confirmation in larger lesion-specific comparative studies before any advantage over conventional framing techniques—in efficacy or procedural efficiency—can be established.

## Figures and Tables

**Table 1:** Baseline patient and lesion characteristics.

Total Patients/Total Lesions, n	13/14
Sex, n (%)	
Female	10 (76.9)
Male	3 (23.1)
Age (years), M ± SD [Range]	60.62 ± 11.22 [42–79]
Disease Category, patients (lesions)	
PAVM	7 (7)
SAA	5 (6)
HAP	1 (1)

*Abbreviations:* PAVM, pulmonary arteriovenous malformation; SAA, splenic artery aneurysm; HAP, hepatic artery pseudoaneurysm; M, mean; SD, standard deviation.

**Table 2:** Lesion characteristics and embolization outcomes.

	PAVM (n = 7)	SAA (n = 6)	HAP (n = 1)	Overall (n = 14)
Diameter (mm) M ± SD [Range]				
Feeding Artery/Inflow	4.20 ± 1.47 [2.8–6.3]	4.34 ± 1.82 [3.1–7.5]	2.9	—
Draining Vein/Outflow	5.30 ± 1.85 [3.0–8.3]	3.17 ± 0.44 [2.6–3.9]	2.8	—
Venous Sac/Sac	7.49 ± 1.56 [5.0–9.1]	27.67 ± 22.18 [6.0–63]	22	—
3D Coil Role, n (%)				
Both (Framing & Packing)	7 (100)	3 (50)	1 (100)	11 (78.6)
Framing only	0	3 (50)	0	3 (21.4)
Coil Counts M ± SD [Range]				
3D Coils	3.00 ± 0.82 [2–4]	3.33 ± 1.75 [1–6]	2	3.07 ± 1.27 [1–6]
Total Coils	6.71 ± 1.11 [5–8]	9.67 ± 5.54 [2–18]	7	8.00 ± 3.82 [2–18]

*Abbreviations:* PAVM, pulmonary arteriovenous malformation; SAA, splenic artery aneurysm; HAP, hepatic artery pseudoaneurysm; 3D, three-dimensional; M, mean; SD, standard deviation.

**Table 3:** Procedural and clinical outcomes and Complications.

Outcome	n/N	% (95% CI)
Technical success	14/14	100 (78.5–100)
Angiographic success, overall	13/14	92.9 (68.5–98.7)
PAVMs	6/7	85.7 (48.7–97.4)
SAAs	6/6	100 (61.0–100)
HAP	1/1	100 (20.7–100)
Clinical success, overall	12/13	92.3 (66.7–98.6)
PAVMs	5/6	83.3 (43.6–97.0)
SAAs	6/6	100 (61.0–100)
HAP	1/1	100 (20.7–100)
Complications		
PES	6/13	46.2 (23.2–70.9)
Splenic Infarction (SAAs)	5/5	100 (56.6–100)
Major Complications	0/13	—

Complications were assessed on a per-patient basis (PAVM, n = 7; SAA, n = 5; HAP, n = 1; overall, n = 13). *Abbreviations:* PAVM, pulmonary arteriovenous malformation; SAA, splenic artery aneurysm; HAP, hepatic artery pseudoaneurysm; CI, confidence interval.

## Data Availability

The data presented in this study are available on request from the corresponding author due to patient privacy and ethical restrictions.

## Supplementary Materials

The following supporting information can be downloaded at: https://www.mdpi.com/article/10.3390/medicina62071298/s1, Figure S1: Flow chart of patient selection.

## Abbreviations

The following abbreviations are used in this manuscript:

3D	Three-Dimensional
PAVMs	Pulmonary arteriovenous malformations
SAAs	Splenic artery aneurysms
HAP	Hepatic artery pseudoaneurysm
PES	Post-embolization syndrome
CT	Computed Tomography
