# Supplementary figures and images for: Feasibility and Effectiveness of 3D Coil Framing in the Embolization of Pulmonary Arteriovenous Malformations and Visceral Artery Aneurysms and Pseudoaneurysms
Source: Medicina (Kaunas). 2026 Jul 5;62(7):1298. doi: 10.3390/medicina62071298 (PMC13413707; doi:10.3390/medicina62071298)

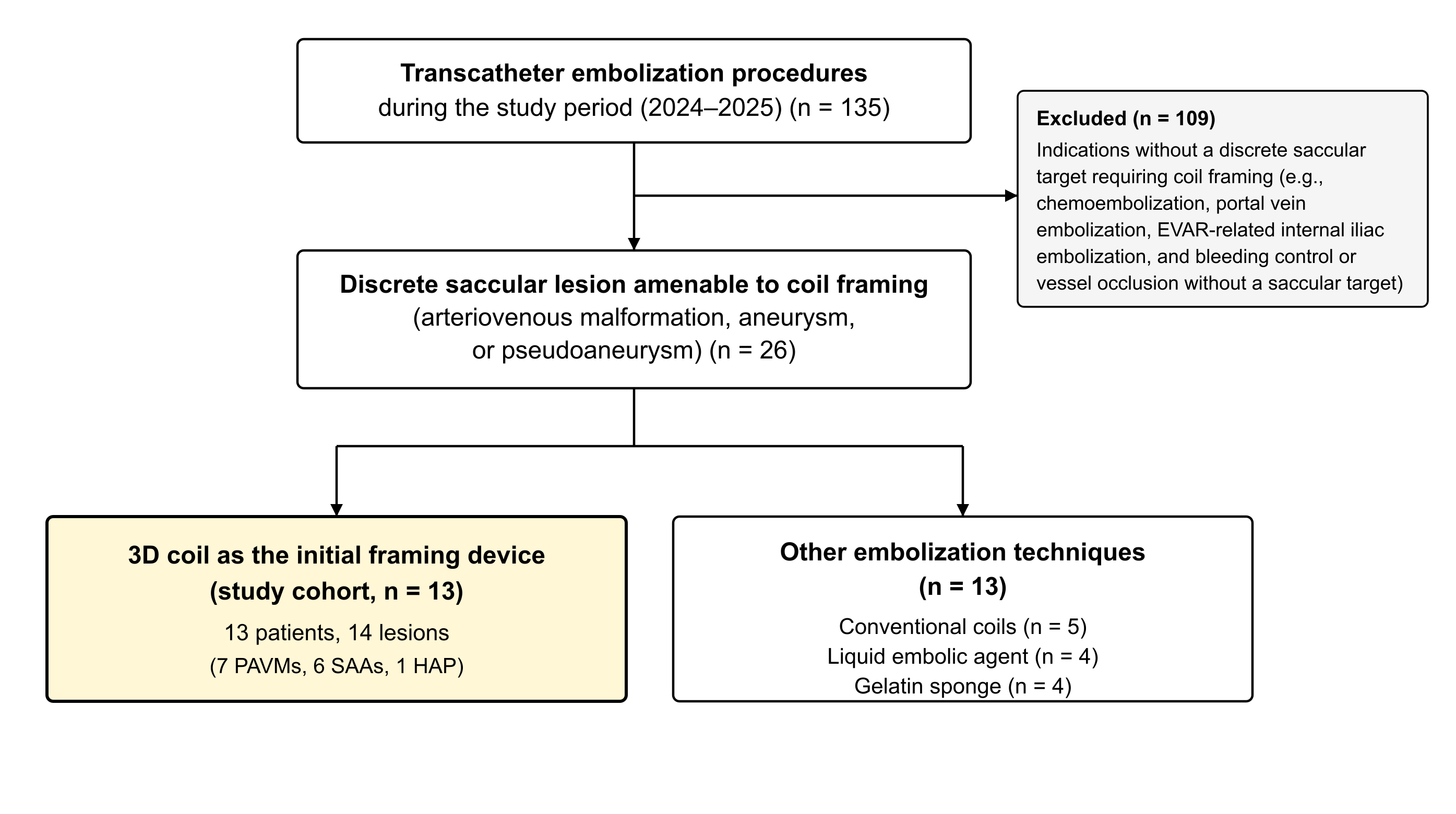

Supplement: Supplementary file 1 [file medicina-62-01298-s001.zip › medicina-4390445-supplementary Figure S1.png]
